# Relative Deprivation and Game Addiction in Left-Behind Children: A Moderated Mediation

**DOI:** 10.3389/fpsyg.2021.639051

**Published:** 2021-06-03

**Authors:** Banglin Yang, Ge Cai, Cancan Xiong, Jin Huang

**Affiliations:** ^1^Faculty of Education, East China Normal University, Shanghai, China; ^2^Collaborative Innovation Center of Assessment Towards Basic Education Quality, Beijing, China; ^3^Sanmenxia Polytechnic, College of Applied Engineering, Henan University of Science and Technology, Sanmenxia, China

**Keywords:** relative deprivation, deviant peer affiliation, beliefs about adversity, game addiction, left-behind children

## Abstract

Previous findings show that relative deprivation has a profound influence on game addiction, but the potential mediating and moderating mechanisms are unclear, especially for left-behind children. The present study therefore examined the relationship between relative deprivation and game addiction, the mediating effect of deviant peer affiliation, and the moderating effect of beliefs about adversity in a sample of left-behind children. A total of 952 left-behind children (mean age = 13.67 years, *SD* = 1.34) participated in this study. The participants anonymously completed a battery of questionnaires, including the Relative Deprivation Scale, the Deviant Peer Affiliation Scale, the Beliefs about Adversity Scale, the Game Addiction Scale, and demographic variables. After controlling for gender, left-behind category, and socioeconomic status, the moderated mediation model showed that (a) relative deprivation significantly and positively predicted game addiction in left-behind children; (b) The mediation analysis showed that the positive association between relative deprivation and game addiction in left-behind children was mediated by deviant peer affiliation; (c) Beliefs about adversity moderated the association between relative deprivation and deviant peer affiliation and were weaker for left-behind children with higher levels of beliefs about adversity, consistent with the risk-buffering model, but the relationship between relative deprivation and game addiction was stronger for left-behind children with higher levels of beliefs about adversity, consistent with the reverse risk-buffering model. These findings have crucial implications for the prevention and intervention of game addiction in left-behind children.

## Introduction

According to the 43rd *Statistical Report on Internet Development in China*, adolescents aged between 12 and 16 years are a high-risk group for game addiction. Game addiction refers to excessive and obsessive-compulsive gaming behavior over which one has no self-control; it usually manifests as the generation of physical and mental dependence and social function impairment caused by the excessive playing of online games (Rehbein and Baier, 2013). Compared with non-left-behind children, parents who work out of town for long periods create a situation in which left-behind children are more susceptible to game addiction (Wei and Luo, 2017). A common feature of left-behind children is that one or more of their parents work out of town continuously for 3 months or longer, and they are left at home to study under the guardianship or management of a single parent or the grandparents (Fan and Lu, 2020). As of August 2018, the number of left-behind children in China reached 6.97 million, and the proportion of left-behind children in the junior high school stage accounted for 19.5% (Ministry of Civil Affairs of the People’s Republic of China, 2018). Due to the presence of problems such as longer spatial distances from parents and insufficient educational energy, this special group of left-behind children is more prone to the emergence of a series of “left-behind children syndromes,” such as weariness with school and spending too much time at Internet cafes (Wang et al., 2019), and they are more prone to escape from reality through gaming, thereby forming a game addiction (Tang et al., 2018). With the gradual increase in the number of left-behind children and the increasingly serious problem of game addiction, an inquiry into the influencing factors and mechanism of action of game addiction in left-behind children has important practical significance.

### Relative Deprivation and Game Addiction

Relative deprivation refers to an individual’s subjective experience of perceiving that she/he is in a negative situation by making an upward social comparison with a reference target object and then generating negative emotions such as dissatisfaction and anger (Greitemeyer and Sagioglou, 2019). According to the classic theory on relative deprivation, members of vulnerable groups in society experience feelings of relative deprivation in basic rights in the course of comparing themselves with others, and such a sense of relative deprivation generates negative influences on their psychological adjustment, thereby inducing negative emotions and problem behaviors (Smith et al., 2012; Greitemeyer and Sagioglou, 2017; Lin and Liu, 2020). On one hand, online games can make up for an individual’s unmet psychological needs due to relative deprivation. Online games can provide individuals with the possibility of temporarily masking their real identities and establishing new identities and a new status (Callan et al., 2015). By creating a virtual avatar similar to the ideal self, the individual can temporarily reduce the tension caused by the difference between reality and the ideal self, and meet basic psychological needs (Heng et al., 2018). On the other hand, online games can also become a “safe haven” for individuals to escape from the pressure of reality due to relative deprivation (Kwon et al., 2011). Because of their availability and the associated stimulation and flow state, online games can easily become a way for individuals to escape the pressure of reality. To meet psychological needs and vent the negative emotions induced by relative deprivation, left-behind children are inclined to establish new identities through online gaming to escape the pressures of reality; subsequently, an addiction to video games can develop over time. It has been found that the perception of relative deprivation causes emotions of dissatisfaction and anger in individuals, leading to problem behaviors such as Internet addiction (Ding et al., 2020). Recent research has discussed the influence of parenting styles, the school climate, and gratitude on game addiction in left-behind children (Wei et al., 2015; Yang and Huang, 2020). However, as a kind of social psychological variable reflecting the interaction between macroenvironmental factors and micro individual factors, relative deprivation is rarely studied. Left-behind children are a typical vulnerable group, and when they compare themselves with non-left-behind children or other groups of children around them, a sense of relative deprivation is prone to be generated psychologically (Xiong and Liu, 2020). Therefore, confirmation that relative deprivation is a risk factor for game addiction in left-behind children will undoubtedly provide a new perspective for understanding its formation and development.

### Deviant Peer Affiliation as a Mediator

Deviant peer affiliation refers to affiliations with peers who violate school rules, social norms, and even laws (Rudolph et al., 2014). Deviant peer affiliation may have a substantial role in mediating the relationship between relative deprivation and game addiction. The meeting of basic psychological needs is one of the proposed mechanisms used to explain the association between relative deprivation and game addiction *via* deviant peer affiliation. According to self-determination theory, individuals are born with basic psychological needs, including autonomy, competence, and belonging (Deci and Ryan, 2012). When individuals experience relative deprivation, injustice, and unsatisfied needs in real life, they are prone to maladjustment or the choosing of different pathways to satisfy their needs (Callan et al., 2008). Deviant peers usually have a higher social status and more autonomy and seem to be less restricted by rules (Su et al., 2016). Hence, when left-behind children perceive relative deprivation, they think they are in a disadvantaged status; their basic psychological needs, such as status needs, are often unmet, and they are more willing to make friends with deviant peers to satisfy status and autonomy needs. Therefore, relative deprivation may be a risk factor for deviant peer affiliation.

In addition, left-behind children who have many deviant peer affiliations are more likely to develop game addiction. After entering adolescence, the influence of peer relationships on adolescents gradually increases and becomes one of the most critical ecosystems affecting their development (Yu et al., 2018). According to peer norms theory, peer group attitudes and norms generate pressure on individuals, who can only obtain group acceptance and reinforcement by maintaining problem behaviors; otherwise, they will be marginalized by the peer group or even kicked out of the group (Blanton and Burkley, 2008). Left-behind children may also be subjected to peer pressure and be unwilling to refuse the invitation of deviant peers, which increases the risk of playing video games. When left-behind children who are addicted to Internet games make friends, the process transitions from “selection” to “socialization,” and the two are interwoven and mutually reinforcing; for example, playing online games together can help deviant peers (Ping et al., 2017). Studies have shown that deviant peer affiliation is a proximal factor for Internet gaming addiction (Tian et al., 2019). For instance, a study of 833 Chinese adolescents revealed that deviant peer affiliation positively relates to game addiction (Zhu et al., 2015). According to theoretical and empirical analyses, it is reasonable to speculate that deviant peer affiliation mediates the positive association between relative deprivation and game addiction in left-behind children (H1).

### Beliefs About Adversity as a Moderator

**FIGURE 1:**
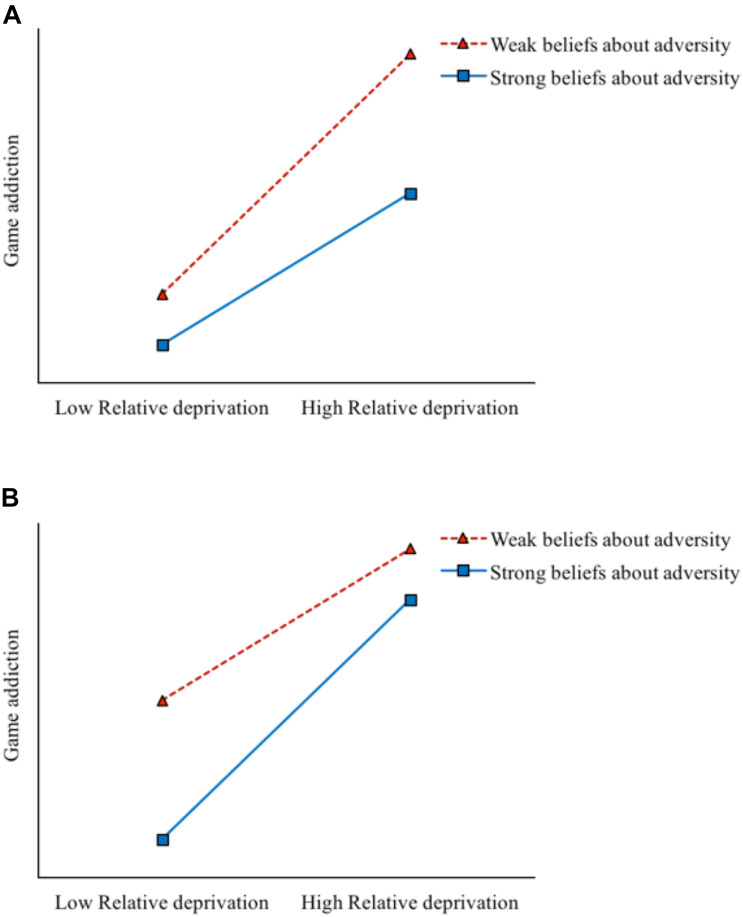
Two different moderating models of beliefs about adversity on the relation between relative deprivation and left-behind children’s game addiction. **(A)** Risk-buffering model. **(B)** Reverse risk-buffering model.

Importantly, although relative deprivation generates effects on game addiction in left-behind children, not every left-behind child will become addicted to games or develop deviant peer affiliations. Similar to the differential susceptibility hypothesis, the interaction between an individual’s environment and his/her own features (such as beliefs) will affect his/her psychological adaptation, possibly because certain individual or environmental factors play protective roles, which could buffer the negative influence of relative deprivation (Belsky and Pluess, 2009). In recent years, beliefs about adversity as a concept of Chinese cultural characteristics have gradually attracted the attention of researchers. Beliefs about adversity refer to an individual’s understanding of the nature of adversity, including the causes and results of adversity and how to cope with it (Shek, 2005; Yunong et al., 2014). Individuals who hold positive beliefs about adversity can seek positive meaning in adversity usually through an understanding and acceptance of it (Wang et al., 2020). Studies have shown that beliefs about adversity are a protective factor for positive adaptation (a sense of integrity, positive emotions, and so on) and against problem behaviors (aggression, depression, and so on) in adolescents (Li et al., 2018). For instance, a study of 776 middle school students reported that beliefs about adversity played a moderating role between negative alterations in mood and cognition symptoms and Internet addiction (Yang et al., 2020). Therefore, beliefs about adversity, as a protective factor, may moderate (i.e., buffer or exacerbate) the size of the role (deviant peer affiliation and game addiction) of a risk factor (relative deprivation). Notably, adolescents will inevitably have contact with deviant peers in the growth environment (Wang et al., 2017). However, if left-behind children hold strong beliefs about adversity, the negative influence of deviant peers may be reduced to a certain extent, and they may be more likely to engage in positive behavior rather than being forced by peer pressure to increase game use behaviors, thereby reducing the risk of game addiction. Therefore, beliefs about adversity (individual factor) may also moderate the size of the role (game addiction) of deviant peer affiliation (peer factor). Two possible models for the process of beliefs about adversity playing a moderating role include the “risk-buffering model” and the “reverse risk-buffering model” (Peng et al., 2019). Under the risk-buffering model, beliefs about adversity can buffer the negative influence of relative deprivation and deviant peer affiliation, especially in a high-risk situation; that is, when relative deprivation and deviant peer affiliation are higher, the protective role of beliefs about adversity is greater, which can benefit left-behind children in a high-risk situation (see Figure 1A). In contrast, under the “reverse risk-buffering model,” in a high-risk situation, that is, when relative deprivation and the level of deviant peer affiliation are higher, the protective role of beliefs about adversity will be weakened (see Figure 1B). According to theoretical and empirical analyses, we hypothesized that beliefs about adversity moderate the relationship between relative deprivation and game addiction (H2); an exploratory analysis was conducted using specific moderating models, but no specific assumptions were made.

### The Present Study

**FIGURE 2:**
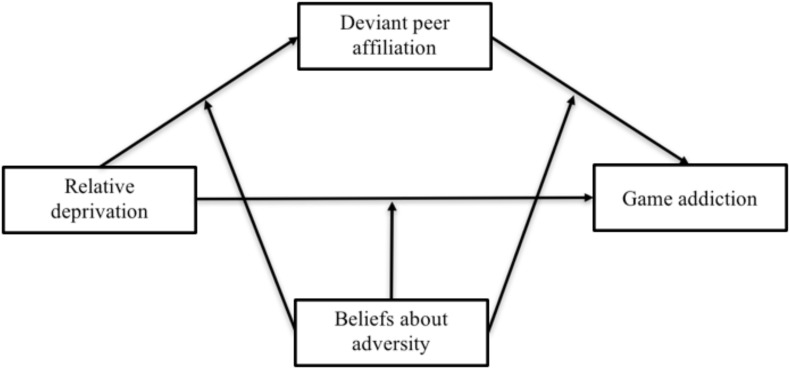
The moderated mediation model.

A simple mediation or moderation model can provide limited valuable information. To address the gaps in the literature, we constructed a moderated mediation model to examine the two aforementioned mechanisms simultaneously to yield more information (Cummings et al., 2000). In summary, we constructed a moderated mediation model (see Figure 2) that specifies a pathway between relative deprivation and game addiction in left-behind children *via* deviant peer affiliation and considers the moderating role of beliefs about adversity in the direct mediation process. We hope this study will provide a basis for the scientific prevention and treatment of game addiction in left-behind children.

## Materials and Methods

### Participants

This study included 952 left-behind children from four junior high schools in Taiqian, which is an economically depressed rural area with a large number of emigrating workers. The average age of the left-behind children was 13.67 (*SD* = 1.34), with an age range of 11–17 years old. Of the 952 left-behind children, 494 participants were girls (51.89%), and 458 participants were boys (48.11%). Regarding the left-behind category, these left-behind children were divided into both-parent-migrant (*N* = 383, 40.23%) and single-parent-migrant (*N* = 569, 59.77%) groups (Fan et al., 2018).

### Measure

#### Relative Deprivation

Relative deprivation was assessed with the Relative Deprivation Scale developed by Ma (2012). This questionnaire contains four items, such as “I feel like people around me always steal my thunder.” Left-behind children rated each item on a six-point scale ranging from 1 (*strongly disagree*) to 6 (*strongly agree*). The average score of the four items was calculated, with a higher total average score indicating higher degrees of relative deprivation. In this study, the internal reliability of the relative deprivation instrument was excellent (α = 0.93).

#### Deviant Peer Affiliation

Deviant peer affiliation was assessed with the Deviant Peer Affiliation Scale, which has been indicated to be suitable for Chinese children (Tian et al., 2019). The eight-item scale measures how many of the respondent’s friends engage in deviant behaviors, including cheating on examinations and problematic Internet use. Left-behind children rated each item on a five-point scale ranging from 1 (*none*) to 5 (*all*). The average score of the eight items was calculated, and a higher total average score indicated a higher degree of deviant peer affiliation. In this study, Cronbach’s alpha was excellent (α = 0.91).

#### Beliefs About Adversity

Beliefs about adversity were measured with the Beliefs about Adversity Scale developed by Shek (2005). This questionnaire contains nine items, such as “[chī de kǔ zhōng kǔ, fāng wéi rén shàng rén] (If you wish to be the best man, you should be willing to suffer the bitterest of the bitter).” Left-behind children rated each item on a six-point scale ranging from 1 (*strongly disagree*) to 6 (*strongly agree*). The average score of the nine items was calculated, with a higher total average score indicating a higher degree of beliefs about adversity. This scale has been demonstrated to be suitable for Chinese children (Li et al., 2018). In this study, Cronbach’s alpha was excellent (α = 0.93).

#### Game Addiction

Game addiction was measured with the Game Addiction Scale developed by Li (2013). This measure includes seventeen items related to five personality dimensions: physiological dependence, emotional dependency, cognitive dependency, behavioral dependency, and functional damage. A sample item is “I used to have trouble sleeping because of playing games.” Left-behind children rated each item on a six-point scale ranging from 1 (*not at all true*) to 5 (*all true*). The average score of the nine items was calculated, with a higher total average score indicating a higher degree of game addiction. This scale has been demonstrated to be suitable for Chinese left-behind children (Yang et al., 2020). In this study, Cronbach’s alpha was excellent (α = 0.87).

#### Demographic Variables

Prior studies have shown that demographic variables such as gender, age, left-behind category, and socioeconomic status are correlated with game addiction in left-behind children (Wang et al., 2019; Yang et al., 2020). Therefore, these variables were controlled as covariates in our study. Gender was dummy coded as 0 (female) and 1 (male). The left-behind category was dummy coded as 0 (both-parent-migrant) and 1 (single-parent-migrant). In addition, because of the particularity of left-behind children, we used the education level of the parents and family financial pressure to represent the socioeconomic status of left-behind children (Zhao et al., 2017). The education level of the parents consisted of “elementary school or below, junior middle school, high school, and bachelor’s degree (1–5 points).” Family financial difficulties were surveyed with the Family Financial Difficulties Scale, which includes four items (Wang et al., 2010). Left-behind children reported the frequency of family financial stress in the past year on a five-point scale ranging from 1 (*never*) to 5 (*always*). In our study, Cronbach’s alpha was excellent (α = 0.91). Finally, the total score of the perceived economic pressure and the education level of the parents were taken as indicators of the socioeconomic status of the left-behind children, with higher scores representing higher socioeconomic statuses.

### Data Analysis

The data were analyzed using IBM SPSS version 24.0 and Hayes’s (2013) PROCESS macro. We calculated the descriptive statistics and correlations of the main study variables. PROCESS macro (Model 4) was used to investigate the mediating role of deviant peer affiliation, and PROCESS macro (Model 59) was used to investigate the moderating effect of beliefs about adversity. Furthermore, we handled the missing data by mean imputation, and we used Harman’s single-factor test to determine the presence of common method bias.

## Results

### Common Method Bias

The data for this study were based on self-reports from left-behind children. Therefore, this study may have common method bias (Podsakoff and Organ, 1986). We used Harman’s one-factor test to determine the presence of common method bias, and the results show that the first principal factor explained 19.27% of the variance (<40%), which illustrates that common method bias is unlikely to be a serious problem.

### Preliminary Analyses

Table 1 presents the descriptive statistics (means and standard deviations) and correlations for the main study variables. Overall, relative deprivation and deviant peer affiliation were positively associated with game addiction in the left-behind children (*r* = 0.61, *p* < 0.01 and *r* = 0.55, *p* < 0.01, respectively), indicating that relative deprivation and deviant peer affiliation were game addiction risk factors. Beliefs about adversity were negatively associated with game addiction (*r* = −0.54, *p* < 0.01). In addition, relative deprivation was positively associated with deviant peer affiliation (*r* = 0.51, *p* < 0.01), whereas relative deprivation was negatively associated with beliefs about adversity (*r* = −0.49, *p* < 0.01). Finally, deviant peer affiliation was negatively associated with beliefs about adversity (*r* = −0.35, *p* < 0.01).

**TABLE 1:** Means, standard deviations, and correlations of the main study variables.

**Variables**	**1**	**2**	**3**	**4**	**5**	**6**	**7**	**8**
1. Age	–							
2. Gender	0.01	–						
3. LBC	0.07	−0.15**	–					
4. SES	0.02	0.02	0.21**	–				
5. RD	−0.23	0.08	−0.20**	−0.35**	–			
6. DPA	0.17**	0.16**	−0.21**	−0.28**	0.51**	–		
7. BAA	0.09*	0.07	0.38**	0.04	−0.49**	−0.35**	–	
8. GA	0.05	0.03	−0.32**	−0.34**	0.60**	0.54**	−0.53**	–
*M*	0.48	0.41	3.49	13.67	3.87	3.30	2.95	3.12
*SD*	0.50	0.49	1.04	1.34	0.78	0.67	0.89	0.93

*Gender was dummy coded such that 0 = female and 1 = male; left-behind category was dummy coded as 0 (both-parent-migrant) and 1 (single-parent-migrant).*

*RD = relative deprivation, DPA = deviant peer affiliation, BAA = beliefs about adversity, GA = game addiction.*

N = 952. *p < 0.05 and **p < 0.01.

### Testing for the Mediating Role of Deviant Peer Affiliation

**FIGURE 3:**
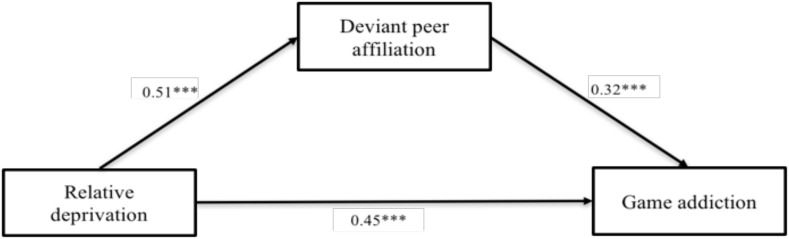
The mediation of deviant peer affiliation. The link between relative deprivation and left-behind children’s game addiction was mediated by deviant peer affiliation. The path values are the path coefficients. Gender, left-behind category and socioeconomic status were controlled during this analysis. ^∗∗∗^*p* < 0.001.

PROCESS macro (Model 4) was used to investigate the mediating role of deviant peer affiliation. As illustrated in Figure 3, after controlling for gender, left-behind category, and socioeconomic status, relative deprivation was positively associated with deviant peer affiliation (β = 0.51, *p* < 0.001), which in turn was positively associated with game addiction (β = 0.32, *p* < 0.001). In addition, the direct association between relative deprivation and game addiction was also significant (β = 0.45, *p* < 0.001), which indicated that deviant peer affiliation mediated the relationship between relative deprivation and externalized problem behaviors (indirect effect = 0.166). The mediating effect accounted for 26.67% of the total effect of relative deprivation on game addiction.

### Testing for Moderated Mediation

PROCESS macro (Model 59) was used to investigate the moderating effect of beliefs about adversity. More specifically, we estimated the parameters for two regression models. In Model 1, we estimated the moderating role of beliefs about adversity in the relationship between relative deprivation and deviant peer affiliation. In Model 2, we estimated the moderating role of beliefs about adversity in the relationships between relative deprivation and game addiction and between deviant peer affiliation and game addiction. The specifications of the two models are shown in Table 2.

**TABLE 2:** Testing the moderated mediation effects of relative deprivation on left-behind children’s game addiction.

**Model**	**Predictors**	***Coeff.***	***SE***	***t***	**95% *CI***
Model 1 (DPA)	Gender	–0.04	0.05	−0.61	[−0.14, 0.08]
	LBC	–0.15	0.06	−2.41***	[−0.27, −0.03]
	SES	–0.04	0.03	−1.48	[−0.08, 0.01]
	RD	0.46	0.03	14.16***	[0.39, 0.52]
	BAA	–0.10	0.03	−3.19***	[−0.17, −0.04]
	RD × BAA	–0.11	0.02	−4.20***	[−0.15, −0.05]
	*R*^2^	0.29			
	*F*	78.38			
Model 2 (GA)	Gender	–0.09	0.03	−3.18***	[−0.19, −0.04]
	LBC	–0.15	0.02	−4.85***	[−0.34, −0.07]
	SES	–0.12	0.03	−4.41***	[−0.27, −0.03]
	RD	0.33	0.03	11.53***	[0.27, 0.39]
	BAA	–0.24	0.03	−8.71***	[−0.30, −0.19]
	RD × BAA	0.08	0.02	3.88***	[0.04, 0.13]
	DPA	0.29	0.03	10.61***	[0.23, 0.34]
	RD × BAA	0.01	0.02	0.23	[−0.04, 0.06]
	*R*^2^	0.53			
	*F*	153.96			

Left-behind category = LBC, Socioeconomic status = SES, RD = relative deprivation, DPA = deviant peer affiliation, BAA = beliefs about adversity, GA = game addiction. *N* = 952. ***p < 0.001.

**FIGURE 4:**
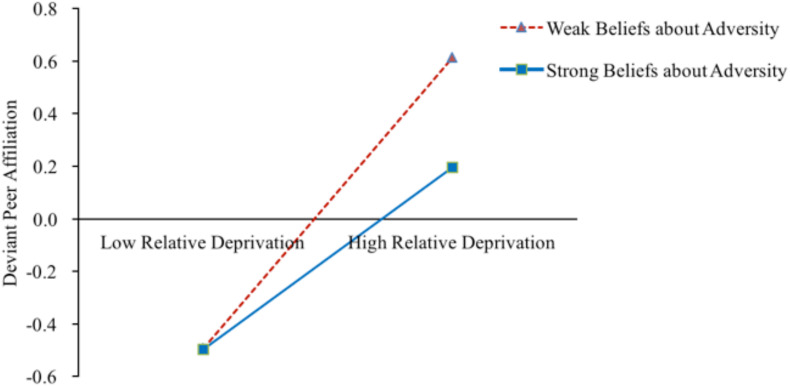
Conditional effect of left-behind children’s deviant peer affiliation as a function of relative deprivation and beliefs about adversity. *M* ± 1*SD* of beliefs about adversity. *N* = 952.

**FIGURE 5:**
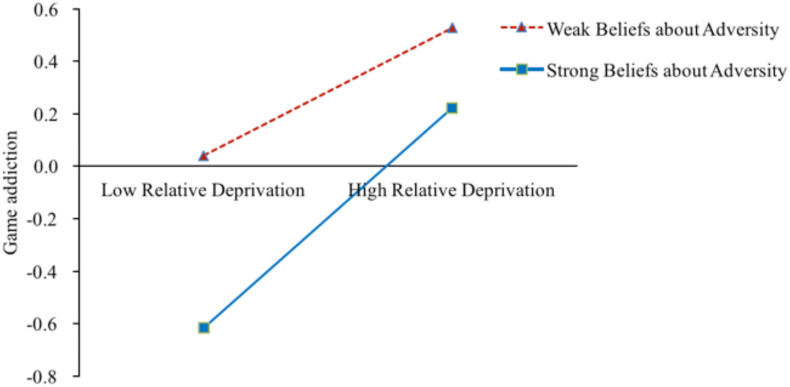
Conditional effect of left-behind children’s game addiction as a function of relative deprivation and beliefs about adversity. *M* ± 1*SD* of beliefs about adversity. *N* = 952.

As shown in Table 2, after gender, left-behind category, and socioeconomic status were controlled for, Model 1 showed that relative deprivation was significantly and positively correlated with deviant peer affiliation (β = 0.46, *p* < 0.001). Furthermore, relative deprivation interacted with beliefs about adversity and was negatively correlated with deviant peer affiliation (β = −0.11, *p* < 0.001). The simple slopes analysis indicated that for left-behind children with stronger beliefs about adversity (*M*+1*SD*), higher levels of relative deprivation were associated with higher levels of game addiction (*B*_*simple*_ = 0.35, *p* < 0.001). However, for left-behind children with weaker beliefs about adversity (*M*−1*SD*), the effect of relative deprivation on deviant peer affiliation was much weaker (*B*_*simple*_ = 0.56, *p* < 0.001) (see Figure 4). Hence, relative deprivation was a much stronger predictor of deviant peer affiliation for left-behind children with lower levels of beliefs about adversity, consistent with the risk-buffering model. Model 2 indicated that relative deprivation was significantly and positively correlated with game addiction (β = 0.33, *p* < 0.001). In addition, relative deprivation interacted with beliefs about adversity related to game addiction (β = 0.08, *p* < 0.001). The simple slopes analysis indicated that for left-behind children with weaker beliefs about adversity (*M*−1*SD*), higher levels of relative deprivation were associated with higher levels of game addiction (*B*_*simple*_ = 0.25, *p* < 0.001). However, for left-behind children with stronger beliefs about adversity (*M*+1*SD*), the effect of relative deprivation on game addiction was much weaker (*B*_*simple*_ = 0.41, *p* < 0.001) (see Figure 5). Hence, relative deprivation was a much stronger predictor of game addiction for left-behind children with higher levels of beliefs about adversity, consistent with the reverse risk-buffering model. In addition, deviant peer affiliation was significantly and positively correlated with game addiction (β = 0.30, *p* < 0.001). However, beliefs about adversity did not moderate this indirect relationship (β = 0.01, *p* > 0.05).

## Discussion

The positive predictive role of relative deprivation on game addiction in adolescents has been confirmed in several studies (Smith et al., 2012; Ding et al., 2020). However, few studies have explored the potential mediating and moderating mechanisms; and even fewer studies have considered left-behind children, a vulnerable group, as the object of study. In this study, a moderated mediation model was constructed to clarify through what (mediating role of deviant peer affiliation) and under what conditions (moderating role of beliefs about adversity) relative deprivation was positively correlated with game addiction in left-behind children.

### Mediating Mechanism of Deviant Peer Affiliation

Although a previous study confirmed relative deprivation as a risk factor for addictive behavior in adolescents (Lin and Liu, 2020), our study is the first empirical study to demonstrate that deviant peer affiliation plays a mediating role between relative deprivation and game addiction in left-behind children. Meanwhile, this result extends self-determination theory, which deepens our understanding by applying it to the process between relative deprivation and game addiction via deviant peer affiliation.

First, relative deprivation was significantly and positively correlated with deviant peer affiliation in left-behind children. The basic psychological needs, such as relationship, ability, and autonomy needs, of adolescent left-behind children are growing rapidly. However, when they perceive a high degree of relative deprivation, their psychological needs are not met, and they easily develop a mentality of insensitivity and laissez-faire. Left-behind children subsequently tend to evade the pressure of reality, pursue peer relationships with higher autonomy, and befriend more deviant peers to obtain basic psychological satisfaction (Chen et al., 2015). Furthermore, left-behind children with high degrees of relative deprivation are more likely to experience anger/frustration (Zhang et al., 2016), which can lead them to affiliate with deviant peers (Oh and Connolly, 2018) to fulfill their social needs. Additionally, deviant peer affiliation was significantly and positively correlated with game addiction in left-behind children. This finding supports peer norms theory; deviant peers will have a negative impact on left-behind children through social imitation, peer pressure, and various forms of reinforcement. On one hand, deviant peers provide opportunities and support for game addiction in left-behind children, thereby leading to the formation of game addiction through the imitation of the game use behaviors and attitudes of deviant peers (Laible et al., 2016; Wang et al., 2016). On the other hand, to better gain their support and acceptance, left-behind children may be forced by peer pressure to comply with online gaming invitations from deviant peers (Dishion and Tipsord, 2011). Additionally, deviant peer affiliation often relies on online games as a core component of social interaction, and game addiction is often unacceptable to parents, teachers, and many classmates, making it easier to form a very strong emotional connection (Su et al., 2016). In turn, this process strengthens the emotional connection between peers, and deviant peer affiliation plays an “amplifier” role in the development of game addiction in left-behind children.

Therefore, to prevent and correct game addiction in left-behind children, it is necessary to emphasize the negative role of deviant peers to prevent the amplification of game addiction.

### Moderating Mechanism of Beliefs About Adversity

It was found in this study that the indirect effects of relative deprivation–deviant peer affiliation–were moderated by beliefs about adversity. According to the “risk-buffering model,” developing beliefs about adversity can help reduce the risk of deviant peer affiliation resulting from relative deprivation. Under the negative backdrop of relative deprivation, beliefs about adversity are conducive to a correct understanding of adversity in left-behind children and can help them find the true essence of life from adversity and use a more positive and optimistic mentality to pursue meaning in life, thereby reducing the negative influence of relative deprivation (Yang et al., 2020). According to the Shift and Persist Model, if an individual can identify a life goal through adversity, it is easy for him/her to adapt to adversity and strengthen his/her beliefs (Chen and Miller, 2012). According to the relationship between the self-shifting and persistence strategy and beliefs about adversity, when left-behind children suffer negative situations, they are more prone to carry out self-shifting, to calmly accept the current situation, and at the same time, to firmly believe that they have the capability and opportunity to change the current situation if they hold higher beliefs about adversity. Therefore, they are more active in seeking meaning in adversity and view adversity as an opportunity, i.e., “when Heaven is about to place great responsibility on someone, it first exhausts that person’s muscles and bones and exposes him to starvation and poverty,” which is conducive to shaping positive behaviors and ultimately reduces the risk of deviant peer affiliation.

Notably, although beliefs about adversity can moderate the relationship between relative deprivation and game addiction, this protective role has limitations, and research results support the “reverse risk-buffering model.” The reason for these limitations may be that left-behind children are not yet mature in their physical and mental development. When they have negative emotions such as dissatisfaction and anger due to relative deprivation, they lack effective coping strategies. In addition, it is difficult for parents to provide effective support. As a result, they can easily turn to other situations to meet basic psychological needs and relieve negative emotions. Online games are more likely to become the first choice for left-behind children to escape the pressure of reality due to their excitement, ease of use, and accessibility. Meanwhile, online games can allow individuals to establish new avatars in the game world, temporarily eliminating the constraints of reality, and the use of avatars in online games allows individuals to obtain high-level game role experience and status satisfaction (Hsu et al., 2009). In other words, due to the highly traumatic nature of relative deprivation and a desire to meet psychological needs, it may be challenging for left-behind children who experience high degrees of relative deprivation to regard the online game world as an effective way to obtain fairness and satisfaction, which in turn leads to game addiction even with strong beliefs about adversity. In addition, we propose the “shared resource assumption” hypothesis to explain this phenomenon. According to this assumption, various components of human cognitive activities will jointly use limited processing resources, and the execution of one cognitive component will interfere with the execution of another cognitive component (Rawson, 2007). The total amount of development resources possessed by an individual in his/her ecological environment is assumed to be limited, the development of capabilities in various fields is assumed to compete with each other for resources, and limitations are assumed to be more obvious, particularly in situations in which resources are scarce. Specifically, we believe that the psychological resources possessed by left-behind children who have experienced higher degrees of relative deprivation are very limited and that their beliefs about adversity may involve a reallocation of psychological resources in different fields, i.e., a type of limited and incomplete psychological resilience.

Finally, it was also found that beliefs about adversity did not moderate the relationship between relative deviant peer affiliation and game addiction in left-behind children, which suggests that the effect of deviant peer affiliation on left-behind children is stronger, more stable, and less prone to being affected by beliefs about adversity. According to the homogeneous selection viewpoint in social networking theory, left-behind children befriending deviant peers is a self-selected process in which they take the initiative to contact and join deviant groups. Thus, they identify with the beliefs, values, and behavioral patterns of deviant peer groups, and their game addiction behaviors are encouraged and supported by their deviant peers (Van Hoorn et al., 2016). Additionally, under the backdrop of deviant peer relationships, left-behind children’s beliefs about adversity may be satirized or ridiculed by their deviant peers, which can easily create conflicts with the beliefs of the deviant peer groups. Thus, beliefs about adversity can be a psychological pressure instead of a kind of protective psychological resource; the protective role of the level of beliefs about adversity is weakened or even disappears (Zhao et al., 2013).

## Limitations and Future Directions

Although valuable findings were generated in this study, there were also some limitations. First, this study was a cross-sectional study based on questionnaire surveys, and it was difficult to draw causal inferences based on the relationships among the variables. The selected research subjects were left-behind children in junior high school who were in puberty, a stage characterized by rapid physical and mental development and a dynamic and unstable state, which will inevitably lead to uncertainty in the study’s results. A longitudinal study could be performed in the future to collect data at multiple time points to investigate the relationship between the variables. Second, the left-behind children selected in the study all came from rural middle schools in Taiqian County, Henan Province; whether or not the results of this study are more broadly suitable for groups of left-behind children in other regions is unclear In future studies, the areas where left-behind children are located could be expanded to carry out larger-scale surveys. Third, with regard to the effect that relative deprivation has on game addiction in left-behind children, deviant peer affiliation (promotion mechanism) and beliefs about adversity (inhibition mechanism) offer different mechanisms of influence, which implies that a closer connection should exist between the two mechanisms. Due to the research conceptualization and hypotheses in this study, it was impossible to explore the internal mechanism more deeply; because of this uncertainty, future studies could further explore the underlying mechanisms for game addiction in left-behind children.

## Implications for Practice

The study results show that game addiction in left-behind children falls under the joint influence of risk and protective factors, which reveals that we need to use integrated and multivariate thinking to formulate intervention and correction strategies to address this issue. First, relative deprivation has an important effect on game addiction; therefore, reducing relative deprivation is the primary way to reduce game addiction in left-behind children. It is necessary to scientifically guide left-behind children in carrying out adversity attribution while simultaneously improving relative deprivation in left-behind children through cognitive training, psychological interventions, and other means. Second, left-behind children are in a sensitive period with regard to their perception of belongingness among peers; because they yearn to make friends with peers to satisfy their emotional needs, attention should be given to the important role of deviant peer affiliation in the development of game addiction. To this end, schools and parents should pay attention to whether or not left-behind children have made friends with deviant peers, communicate with each other in a timely manner, and help left-behind children resolve confusion and difficulties encountered in peer affiliation. Third, “adversity and transcendence” in traditional Chinese culture can be used to help left-behind children understand and learn to handle adversity and encourage them to strive for progress; however, we should also recognize the limitation of beliefs about adversity. Therefore, every effort must be made to mitigate risk factors to truly benefit the positive development of left-behind children instead of simply exaggerating the protective role of beliefs about adversity. Otherwise, once the power of risk factors exceeds the psychological coping resources of left-behind children, they will bear the “cost of resilience” in the course of their development (Evans et al., 2013).

## Data Availability Statement

The raw data supporting the conclusions of this article will be made available by the authors, without undue reservation.

## Ethics Statement

The studies involving human participants were reviewed and approved by University Committee on Human Research Protection of East China Normal University. Written informed consent to participate in this study was provided by the participants’ legal guardian/next of kin. Written informed consent was obtained from the minor(s)’ legal guardian/next of kin for the publication of any potentially identifiable images or data included in this article.

## Author Contributions

BY: conceptualization, methodology, investigation, formal analysis, and writing–original draft. GC: conceptualization, methodology, supervision, and writing. CX: formal analysis and writing. JH: project administration, supervision, and writing. All authors contributed to the article and approved the submitted version.

## Conflict of Interest

The authors declare that the research was conducted in the absence of any commercial or financial relationships that could be construed as a potential conflict of interest.

## References

[B1] Belsky J. Pluess M. (2009). Beyond diathesis stress: differential susceptibility to environmental influences. *Psychol. Bull.* 135 885–908. 10.1037/a0017376 19883141

[B2] Blanton H. Burkley M. (2008). “Deviance regulation theory: applications to adolescent social influence,” in *Understanding Peer Influence in Children and Adolescents*, eds Prnstein M. J. Dodge K. A. (New York, NY: Guilford), 94–121.

[B3] Callan M. J. Ellard J. H. Shead N. W. Hodgins D. C. (2008). Gambling as a search for justice: examining the role of personal relative deprivation in gambling urges and gambling behavior. *Pers. Soc. Psychol. Bull.* 34 1514–1529. 10.1177/0146167208322956 18723773

[B4] Callan M. J. Shead N. W. Olson J. M. (2015). The relation between personal relative deprivation and the urge to gamble among gamblers is moderated by problem gambling severity: a meta-analysis. *Addict. Behav.* 45 146–149. 10.1016/j.addbeh.2015.01.031 25665918

[B5] Chen E. Miller G. E. (2012). Shift-and-persist strategies why low socioeconomic status isn’t always bad for health. *Perspect. Psychol. Sci.* 7 135–158. 10.1177/1745691612436694 23144651 PMC3491986

[B6] Chen W. Li D. Bao Z. Yan Y. Zhou Z. (2015). The impact of parent-child attachment on adolescent problematic internet use: a moderated mediation model. *Acta Psychol. Sin.* 47 611–623. 10.3724/sp.j.1041.2015.00611

[B7] Cummings E. M. Davies P. Campbell S. B. (2000). *Developmental Psychopathology and Family Process: Theory, Research, and Clinical Implications.* New York, NY: Guilford Press.

[B8] Deci E. L. Ryan R. M. (2012). “Motivation, personality, and development within embedded social contexts: an overview of self-determination theory,” in *The Oxford Handbook of Human Motivation*, ed. Ryan R. M. (Oxford: Oxford University Press), 85–107.

[B9] Ding Q. Zhang Y. Zhou Z. (2020). Relative deprivation and college students’ online flaming: mediating effect of ego depletion and gender difference. *Psychol. Dev. Educ.* 36 200–207. 10.16187/j.cnki.issn1001-4918.2020.02.09

[B10] Dishion T. J. Tipsord J. M. (2011). Peer contagion in child and adolescent social and emotional development. *Annu. Rev. Psychol.* 62 189–214. 10.1146/annurev.psych.093008.100412 19575606 PMC3523739

[B11] Evans G. W. Li D. Whipple S. S. (2013). Cumulative risk and child development. *Psychol. Bull.* 139 1342–1396. 10.1037/a0031808 23566018

[B12] Fan X. H. Fang X. Y. Huang Y. S. Chen F. J. Yu S. (2018). The influence mechanism of parental care on depression among left-behind rural children in China: a longitudinal study. *Acta Psychol. Sin.* 50 1029–1040. 10.3724/SP.J.1041.2018.01029

[B13] Fan X. Y. Lu M. J. (2020). Testing the effect of perceived social support on left-behind children’s mental well-being in mainland China: the mediation role of resilience. *Child. Youth Serv. Rev.* 109:104695. 10.1016/j.childyouth.2019.104695

[B14] Greitemeyer T. Sagioglou C. (2017). Increasing wealth inequality may increase interpersonal hostility: the relationship between personal relative deprivation and aggression. *J. Soc. Psychol.* 157 766–776. 10.1080/00224545.2017.1288078 28139176

[B15] Greitemeyer T. Sagioglou C. (2019). The impact of personal relative deprivation on aggression over time. *J. Soc. Psychol.* 159 664–675. 10.1080/00224545.2018.1549013 30541413 PMC6816473

[B16] Heng S. P. Zhou Z. K. Sun L. J. (2018). The avatar identification in video games. *Adv. Psychol. Sci.* 25 1565–1578. 10.3724/SP.J.1042.2017.01565

[B17] Hsu S. H. Wen M. H. Wu M.-C. (2009). Exploring user experiences as predictors of MMORPG addiction. *Comput. Educ.* 53 990–999. 10.1016/j.compedu.2009.05.016

[B18] Kwon J. H. Chung C.-S. Lee J. (2011). The effects of escape from self and interpersonal relationship on the pathological use of internet games. *Community Ment. Health J.* 47 113–121. 10.1007/s10597-009-9236-1 19701792

[B19] Laible D. Carlo G. Davis A. N. Karahuta E. (2016). Maternal sensitivity and effortful control in early childhood as predictors of adolescents’ adjustment: the mediating roles of peer group affiliation and social behaviors. *Dev. Psychol.* 52 922–932. 10.1037/dev0000118 27228452

[B20] Li B. (2013). *Research on the Establishment of Adolescent Online Game Addiction Questionnaire and Intervention.* Chongqing: ChongQing University.

[B21] Li Q. Zhang W. Zhao J. (2018). The longitudinal associations among grandparent–grandchild cohesion, cultural beliefs about adversity, and depression in Chinese rural left-behind children. *J. Health Psychol.* 26 140–155. 10.1177/1359105318803708 30284920

[B22] Lin Y. Liu Q. X. (2020). Perceived subjective social status and smartphone addiction tendency among Chinese adolescents: a sequential mediation model. *Child. Youth Serv. Rev.* 116:105222. 10.1016/j.childyouth.2020.105222

[B23] Ma A. (2012). Relative deprivation and social adaption: the role of mediator and moderator. *Acta Psychol. Sin.* 44 377–387. 10.3724/sp.j.1041.2012.00377

[B24] Ministry of Civil Affairs of the People’s Republic of China (2018). *Chart**: Data of Rural Left-Behind Children in 2018. Beijing: 2018-09-01.* Available online at: http://www.mca.gov.cn/article/gk/tjtb/201809/20180900010882.shtml

[B25] Oh G. Connolly E. J. (2018). Anger as a mediator between peer victimization and deviant behavior in South Korea: a cross-cultural application of general strain theory. *Crime Delinq.* 65 1102–1122. 10.1177/0011128718806699

[B26] Peng W. Li D. Li D. Jia J. Wang Y. Sun W. (2019). School disconnectedness and adolescent internet addiction: mediation by self-esteem and moderation by emotional intelligence. *Comput. Human Behav.* 98 111–121. 10.1016/j.chb.2019.04.011

[B27] Ping S. Wei Z. Yu C. Sha L. Yang X. Zhen S. (2017). Influence of parental marital conflict on adolescent aggressive behavior via deviant peer affiliation: a moderated mediation model. *J. Psychol. Sci.* 40 1392–1398.

[B28] Podsakoff P. M. Organ D. W. (1986). Self-reports in organizational research: problems and prospects. *J. Manag.* 12:531e544. 10.1177/014920638601200408

[B29] Rawson K. A. (2007). Testing the shared resource assumption in theories of text processing. *Cogn. Psychol.* 54 155–183. 10.1016/j.cogpsych.2006.06.002 16893536

[B30] Rehbein F. Baier D. (2013). Family-, media-, and school-related risk factors of video game addiction. *J. Media Psychol.* 25 118–128. 10.1027/1864-1105/a000093

[B31] Rudolph K. D. Lansford J. E. Agoston A. M. Sugimura N. Schwartz D. Dodge K. A. (2014). Peer victimization and social alienation: predicting deviant peer affiliation in middle school. *Child Dev.* 85 124–139. 10.1111/cdev.12112 23621796 PMC3732545

[B32] Shek D. T. L. (2005). A longitudinal study of Chinese cultural beliefs about adversity, psychological well-being, delinquency and substance abuse in Chinese adolescents with economic disadvantage. *Soc. Indic. Res.* 71 385–409. 10.1007/s11205-004-8029-8

[B33] Smith H. J. Pettigrew T. F. Pippin G. M. Bialosiewicz S. (2012). Relative deprivation: a theoretical and meta-analytic review. *Pers. Soc. Psychol. Rev.* 16 203–232. 10.1177/1088868311430825 22194251

[B34] Su B. Y. Zhang W. Su Q. Yu C. F. (2016). Why parents’ regulation of internet use was ineffective to adolescent problematic online game use? a moderated mediation model. *Psychol. Dev. Educ.* 32 604–613. 10.16187/j.cnki.issn1001-4918.2016.05.11

[B35] Tang W. Wang G. Hu T. Dai Q. Xu J. Yang Y. (2018). Mental health and psychosocial problems among Chinese left behind children: a cross-sectional comparative study. *J. Affect. Disord.* 241 133–141. 10.1016/j.jad.2018.08.017 30121025

[B36] Tian Y. Yu C. Lin S. Lu J. Liu Y. Zhang W. (2019). Sensation seeking, deviant peer affiliation, and internet gaming addiction among Chinese adolescents: the moderating effect of parental knowledge. *Front. Psychol.* 9:2727. 10.3389/fpsyg.2018.02727 30687181 PMC6336697

[B37] Van Hoorn J. Van Dijk E. Meuwese R. Rieffe C. Crone E. A. (2016). Peer influence on prosocial behavior in adolescence. *J. Res. Adolesc.* 26 90–100. 10.1111/jora.12173

[B38] Wang H. Wang Q. Liu X. Gao Y. Chen Z. (2020). Prospective interpersonal and intrapersonal predictors of initiation and cessation of non-suicidal self-injury among Chinese adolescents. *Int. J. Environ. Res. Public Health* 17:9454. 10.3390/ijerph17249454 33348636 PMC7766568

[B39] Wang J. P. Li D. P. Zhang W. (2010). Adolescence’s family financial difficulty and social adaptation: coping efficacy of compensatory, mediation, and moderation effects. *J. Beijing Normal Univ. (Soc. Sci.)* 4 22–32.

[B40] Wang Q. Xiao T. Liu H. Y. Hu W. (2019). The relationship between parental rejection and internet addiction in left-behind children: a moderated mediation model. *Psychol. Dev. Educ.* 35 749–758.

[B41] Wang Y. H. Li D. P. Sun W. Q. Zhao L. Y. Lai X. F. Zhou Y. Y. (2017). Parent-child attachment and prosocial behavior among junior high school students: moderated mediation effect. *Acta Psychol. Sin.* 49 663–679. 10.3724/SP.J.1041.2017.00663

[B42] Wang Y. H. Wu A. M. S. Lau J. T. F. (2016). The health belief model and number of peers with internet addiction as inter-related factors of Internet addiction among secondary school students in Hong Kong. *BMC Public Health* 16:272. 10.1186/s12889-016-2947-7 26983882 PMC4794899

[B43] Wei C. Jin Z. Y. Liu S. Li Z. X. Wang Y. Yu C. F. (2015). Gratitude and online gaming addiction among left-behind children: the mediating effect of perceptions of school climate. *Chinese School Health* 36 1161–1163. 10.16835/j.cnki.1000-9817.2015.08.016

[B44] Wei C. Luo Q. (2017). Stressful life events and internet gaming disorder among left-behind children: the moderating effect of self-esteem. *Educ. Meas. Eval.* 6 45–51.

[B45] Xiong M. Liu R. J. (2020). The relationship between relative deprivation and depression of the left-behind children: the impact of perceived control and belief in a justice world. *J. Fujian Normal Univ. (Philos. Soc. Sci.)* 2 148–157+171–172.

[B46] Yang B. L. Huang J. (2020). Emotional neglect and gaming addiction of rural left-behind children: the moderating roles of beliefs about adversity. *Chinese J. Spec. Educ.* 9 86–93.

[B47] Yang X. Wu X. Qi J. Zhou X. (2020). Posttraumatic stress symptoms, adversity belief, and internet addiction in adolescents who experienced a major earthquake. *Curr. Psychol.* 4. 10.1007/s12144-020-00816-y

[B48] Yu G. L. Zhao F. Wang H. Li S. (2018). Subjective social class and distrust among Chinese college students: the mediating roles of relative deprivation and belief in a just world. *Curr. Psychol.* 39 2221–2230. 10.1007/s12144-018-9908-5

[B49] Yunong H. Hung W. Ngoh T. T. (2014). Associations among Chinese cultural beliefs of adversity, income recovery, and psychological status of wenchuan earthquake survivors. *Soc. Work Ment. Health* 12 343–364. 10.1080/15332985.2014.889061

[B50] Zhang H. Liu M. Tian Y. (2016). Individual-based relative deprivation (IRD) decreases prosocial behaviors. *Motiv. Emot.* 40 655–666. 10.1007/s11031-016-9564-8

[B51] Zhao J. Liu X. Zhang W. (2013). Peer rejection, peer acceptance and psychological adjustment of left-behind children: the roles of parental cohesion and children’s cultural beliefs about adversity. *Acta Psychol. Sin.* 45 797–810. 10.3724/SP.J.1041.2013.00797

[B52] Zhao J. Luan F. Sun P. Xu T. T. Liu X. (2017). Parental cohesion, beliefs about adversity and left-behind children’s positive/negative emotion in rural China. *Psychol. Dev. Educ.* 33 441–448. 10.3724/SP.J.1041.2013.00797

[B53] Zhu J. Zhang W. Yu C. Bao Z. (2015). Early adolescent internet game addiction in context: how parents, school, and peers impact youth. *Comput. Human Behav.* 50 159–168. 10.1016/j.chb.2015.03.079

